# Cardiac events among patients with sarcoma treated with doxorubicin by method of infusion: A real‐world database study

**DOI:** 10.1002/cnr2.1681

**Published:** 2022-07-18

**Authors:** Lee D. Cranmer, Lisa M. Hess, Tomoko Sugihara, Howard G. Muntz

**Affiliations:** ^1^ Division of Medical Oncology, University of Washington Fred Hutchinson Cancer Research Center Seattle Washington USA; ^2^ Global Health Outcomes Eli Lilly and Company Indianapolis Indiana USA; ^3^ Biometrics Syneos Health Morrisville North Carolina USA; ^4^ Oncology Eli Lilly and Company Indianapolis Indiana USA

**Keywords:** anthracycline, cardiotoxicity, doxorubicin, infusion, retrospective, sarcoma, bolus

## Abstract

**Background:**

Administration of doxorubicin by continuous intravenous (CIV) infusion, versus bolus (BOL) administration, has been proposed to mitigate the risk of cardiac events. This study used real‐world data to explore the association between mode of doxorubicin administration and duration of treatment, time‐to‐treatment failure (TTF), and cardiac events.

**Methods:**

Occurrence of cardiac events after initiation of BOL versus CIV doxorubicin for sarcoma in the International Business Machines MarketScan claims database were compared. Duration of doxorubicin treatment, TTF, and time‐to‐first‐cardiac event (TCE) were evaluated using Kaplan–Meier method and unadjusted and adjusted Cox regression models.

**Results:**

A total of 196 patients were included in the BOL group and 399 in the CIV group. In unadjusted analyses, there were significant differences between BOL versus CIV for duration of doxorubicin treatment (median 1.4 vs. 2.1 months, *p* = .002), TTF (median 8.8 vs. 5.6 months, *p* = .002), and TCE (medians not reached, *p* = .03). Adjusting for baseline covariates, only TTF remained significant (hazard ratio: 0.71, 95% confidence interval 0.59–0.86, *p* = .0004), favoring BOL.

**Conclusions:**

While the risk of cardiac complications was higher with BOL in unadjusted analysis, the risk was no longer present in the adjusted analysis. While we cannot draw causal inferences due to the retrospective, nonrandomized study design, these data suggest that replacing BOL with prolonged CIV administration has not been effective as a strategy to mitigate cardiac events, given community standards of oncologic practice.

## 1 BACKGROUND

Since its introduction in the 1970s, doxorubicin and related anthracyclines have played critical roles in the management of a variety of malignancies, including sarcomas. With limited exceptions, doxorubicin remains the backbone of treatment for advanced sarcoma, either as monotherapy or as part of multi‐drug treatment regimens.^1^, ^2^, ^3^, ^4^, ^5^

The clinical utility of anthracycline therapy is limited by dose‐dependent cardiomyopathy.^6^ Increased risk of cardiac events is associated with a variety of clinical and treatment factors. Patient factors include extremes of age, pre‐existing heart disease, female sex, hypertension, and prior receipt of mediastinal irradiation. Treatment factors may include rate of drug administration, and critically cumulative anthracycline dose. Cumulative doses of doxorubicin less than 300 mg/m^2^ generally have a low risk of cardiotoxicity; this represents a threshold value above which institution of cardioprotective measures is justified.^7^ In early studies, the nonlinear relationship between cumulative doxorubicin dosing and development of clinical congestive heart failure suggested a limitation in dosing of 550 mg/m^2^, with a lower limit of 450 mg/m^2^ considered prudent in patients receiving multi‐agent chemotherapy or cardiac irradiation.^4^, ^5^ The lower limit is generally observed in current dosing with doxorubicin.^1^, ^2^, ^3^

Several strategies have been explored to minimize cardiac events. These include limiting total lifetime anthracycline dose,^8^ development of anthracycline derivatives with less cardiotoxicity (e.g. epirubicin, liposomal encapsulation^9^, ^10^, ^11^, ^12^, ^13^), and use of dexrazoxane, a cardioprotective agent.^7^, ^8^ All of these approaches are used variously in the management of patients receiving anthracycline therapy, including sarcoma patients, but none has yet been adopted as a standardized approach.

Altering the manner of drug administration from bolus (BOL) to continuous intravenous infusion (CIV) is another strategy to control anthracycline cardiotoxicity.^14^ The benefit of this approach was hypothesized as being due to lower peak plasma anthracycline levels in those receiving CIV treatment, versus BOL.^15^, ^16^ Progressively longer infusion times (24–96 h) yielded lower peak plasma drug levels, versus short (5 min) BOL administration. Cardiac effects were assessed by serial endomyocardial biopsies, which yielded a pathologic score of cardiotoxicity characteristic of anthracycline effects. At a given cumulative dose of doxorubicin, patients receiving BOL dosing had higher mean pathologic scores than those receiving CIV dosing, and demonstrated a dose‐dependent increase in the pathologic score. In contrast, those receiving CIV had essentially stable pathologic scores with increasing cumulative doxorubicin dose.

The benefit of CIV administration was tested in four randomized trials, published 1989–1991.^9^ ^,^ ^17^, ^18^, ^19^ These studies varied in their sample sizes, target populations (breast/ovarian cancer, sarcoma; adjuvant vs. metastatic disease), cardiac assessment methods, definitions of cardiotoxicity, treatments (doxorubicin alone vs. combination therapy), duration of CIV (6–96 h), and comparison treatments (BOL epirubicin used as comparator in one trial).^9^ Nevertheless, these trials gave a consistent message: CIV administration of doxorubicin was associated with decreased cardiotoxicity. Notably, all four trials made provision to exceed the 450 mg/m^2^ dosing limit noted above, if judged clinically beneficial. A meta‐analysis suggested odds ratios for clinical and sub‐clinical cardiotoxicity of 4.13 (95% CI 1.75–9.72) and 3.04 (95% CI 1.66–5.58), respectively, favoring CIV over BOL treatment.^11^

Despite these data, BOL administration of doxorubicin is commonly used in sarcoma treatment, including in large, randomized clinical trials, most commonly with the caveat of limiting cumulative dosing to 450 mg/m^2^.^1^, ^2^, ^3^ This is equivalent to six cycles of treatment at a standard dose of 75 mg/m^2^. For example, the SARC021 trial permitted investigators, at their discretion, to administer doxorubicin by either BOL or CIV.^3^, ^20^ Doxorubicin was administered by CIV in only 84/640 (13.1%) of those enrolled.^20^ In a *post hoc* analysis, after adjusting for demographic, clinical, prognostic, and treatment factors, there was no difference in either efficacy or adverse events (cardiac, hematologic, or nonhematologic) based on manner of doxorubicin of administration. Cardiac adverse events were associated with cumulative doxorubicin dose. No statistically significant interaction between doxorubicin dose and mode of administration was evident.

Doxorubicin continues to be a critical agent in sarcoma management. However, as presently used, are sarcoma patients being harmed by its logistically simpler BOL administration, rather than CIV? To address this knowledge gap, this study evaluated real‐world data to understand better the relationship between mode of doxorubicin administration (BOL vs. CIV) and cardiac events, as well as between mode of administration and the duration of doxorubicin treatment and time‐to‐treatment failure (TTF).

## 2 METHODS

### 2.1 Data source

The International Business Machines (IBM) MarketScan® (formerly Truven) databases contain de‐identified, Health Insurance Portability and Accountability Act (HIPAA)‐compliant, fully integrated, patient‐level inpatient, outpatient, and drug data from commercial, Medicaid and employer‐sponsored Medicare supplemental plans.^21^ MarketScan databases have been used in over 300 peer‐reviewed articles published in leading journals since 1990. The databases reflect the real‐world healthcare experience of employees, retirees, and dependents covered by the health benefit programs of large employers. Data are collected from approximately 350 different insurance companies and third‐party administrators. Rigorous validation methods are utilized to ensure that claims and enrollment data are complete, accurate, and reliable. The data files contain patient enrollment information (such as basic demographic and health plan data); medical claims, including diagnosis codes (International Classification of Disease, ICD), Current Procedural Terminology (CPT) and Healthcare Common Procedure Coding System (HCPCS) codes; inpatient and outpatient data; provider type and place of service; pharmacy claims, including drugs dispensed and quantity supplied; and healthcare plan and patient co‐payment amounts. Studies using de‐identified databases are not considered human subjects research by the Department of Health and Human Services and are therefore exempted from institutional review board review by maintaining appropriate privacy standards in accordance with HIPAA.^22^ At the time of this analysis, data were available through December 31, 2019.

### 2.2 Cohort identification

Adult patients aged 18 years or older in the MarketScan commercial claims databases were eligible for inclusion if there was evidence of at least two cancer codes for sarcoma (ICD‐9: 171.x or ICD‐10: C49.x) on two different dates on or after January 1, 2008. Additionally, at least two claims for doxorubicin were required for inclusion in this study. Patients with less than 6 months of uninterrupted enrollment in the database were excluded from this analysis. Additionally, patients with at least two outpatient codes or one inpatient code for atrial fibrillation, cardiac arrest, unstable angina, cardiomyopathy, myocardial infarction or congestive heart failure prior to the first claim for doxorubicin were excluded to avoid misattributing pre‐existing conditions for cardiac events relevant to this study.

Patients were considered to be treated for metastatic disease if codes indicating metastatic disease were present ±90 days of the initial doxorubicin infusion. Patients were considered to be treated in the neoadjuvant or adjuvant setting if there were codes for extirpative surgical procedures and if there were no metastatic codes observed within ±90 days of the first doxorubicin administration. All other patients could not be definitively categorized as having been treated in the metastatic or adjuvant setting in the database. Patients who had no evidence of any other systemic therapy agent (chemotherapy, targeted therapy, or biologic therapy) from the first to last infusion of doxorubicin were considered to have received doxorubicin monotherapy. All others were categorized as combination therapy.

The index date for this study was defined as the date on which the first dose of doxorubicin was administered. Patients were assigned to the BOL group if they had short term infusion codes (CPT 96 408, 96 409, 96 411, 96 420 or HCPCS Q0083) on the same day as the doxorubicin claim, without continuous infusion codes. Patients were assigned to the CIV group if they had continuous infusion codes (ICD‐9: 9925; ICD‐10:3E3305; CPT: 96 365, 96 366, 96 379, 96 410, 96 412–96 416, 96 422, 96 425, 96 440, 96 445, 96 446, 96 423 or C8957) without evidence of short‐term infusion codes on the same date. Patients with both sets of codes on the same day were not assigned to either BOL or CIV, and were not included in the primary analyses. Finally, patients were considered to be chemotherapy‐naïve at the index doxorubicin infusion if there was no record of systemic therapy in the database prior to receipt of doxorubicin.

### 2.3 Outcomes evaluated

Time‐to‐event outcomes evaluated included duration of therapy, TTF, and time‐to‐first‐cardiac‐event (TCE) code. Duration of therapy was defined as time from the first claim for doxorubicin through the last claim for doxorubicin, without censoring, and with inclusion of gap periods without doxorubicin treatment. Sensitivity analyses were conducted that considered these gap periods by evaluating duration of therapy up to a 90‐day or greater gap period. The event date for TTF was defined as the number of days from index date to the first of any of the following events^1^: last observation in the database if the observation occurred >4 months from end of available data^2^; start of a new line of therapy based on a new claim for any systemic chemotherapy agent after the last claim of doxorubicin in the database; or^3^ a hospice claim. All patients without events were censored for the TTF analysis at the last observation in the database. The first observation of a cardiac event code was used for the cardiac toxicity time‐to‐event analyses. Additionally, three time periods were evaluated for the observation of the first cardiac event: early (≤1 year of the index date); middle (>1–5 years of the index date) and late period (>5 years after the index date).

### 2.4 Statistical methods

The cumulative number of doxorubicin claims was evaluated regarding cardiac events during the follow up period to determine whether patients with greater number of infusions are more or less likely to have cardiac event codes. A two‐sample *t*‐test was conducted comparing presence or absence of cardiac events and number of doxorubicin claims. Chi square or Fisher's exact tests compared, by BOL or CIV doxorubicin administration, the proportion of patients with cardiac event codes overall, as well as those occurring during the early, middle, or late periods after initiating doxorubicin.

Time‐to‐event analyses (TCE, duration of therapy, and TTF) for the BOL versus CIV groups were conducted using the Kaplan–Meier method and log‐rank test, as well as covariate‐adjusted Cox proportional hazards regression models. Covariates in the adjusted Cox model were selected in a stepwise manner. All variables reaching statistical significance (*p* < .05) were included in the adjusted analyses. Covariates considered included age, sex, comorbidities as measured by the Charlson comorbidity index (CCI),^23^ health insurance plan type, indication for treatment (adjuvant, metastatic, or unknown), number of concomitant prescription drugs, doxorubicin monotherapy versus combination therapy, and additional cancer site codes.

## 3 RESULTS

**FIGURE 1:**
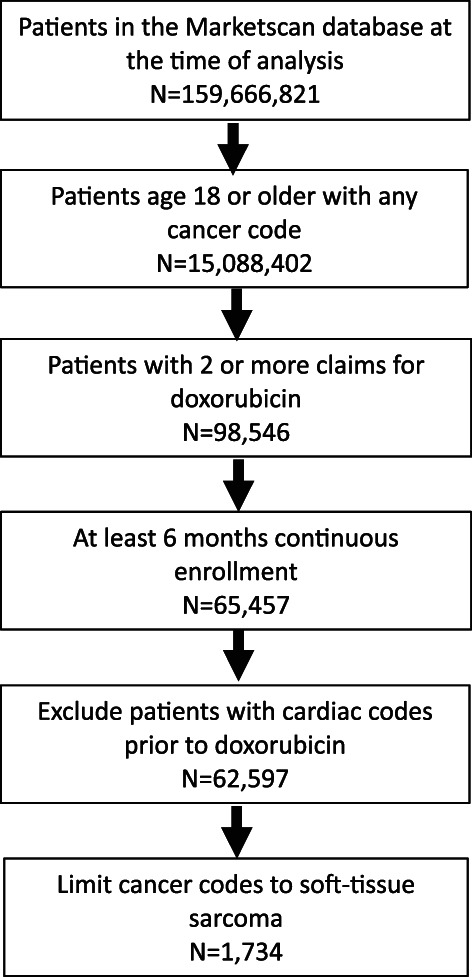
Patient eligibility diagram.

A total of 1734 patients were eligible for inclusion (Figure 1). There were 196 (11.3%) patients identified for the BOL group and 399 (23.0%) in the CIV group. All other patients had multiple treatment administration codes present and could not be definitively assigned to either BOL or CIV DOX. The characteristics of the study cohort are summarized in Table 1. The mean age of patients in the BOL group was 54.9 years (*SD* = 12.7) and in the CIV group mean age was 50.8 years (*SD* = 13.0). Female patients comprised 47.9% of the BOL group and 51.4% of the CIV group. There were similar rates of hypertension (60% in both groups) and total prescription drug burden (mean of 6 concomitant medications) at the time of doxorubicin initiation. There were no differences in duration of follow up, with a median of 11.6 months of follow up (95% CI: 9.6–13.4) for the patients in the BOL subgroup versus 11.6 months (95% CI: 10.2–13.0) for the CIV subgroup.

**TABLE 1:** Characteristics at the time of doxorubicin initiation.

Characteristic	Bolus administration (*N* = 194)	Continuous infusion (*N* = 399)	All other patients (*N* = 1040)
Age, mean (*SD*)	54.9 (12.7)	50.8 (13.0)	47.8 (13.9)
*Sex, n (%)*			
Female	93 (47.9)	205 (51.4)	500 (48.1)
Male	101 (52.1)	194 (48.6)	540 (51.9)
*Geographic region, n (%)*			
North central	57 (29.4)	121 (30.3)	267 (25.7)
North east	43 (22.2)	15 (3.8)	201 (19.3)
South	67 (34.5)	180 (45.1)	426 (41.0)
West	26 (13.4)	80 (20.1)	138 (13.3)
Unknown/missing	1 (0.5)	3 (0.8)	8 (0.8)
*Healthcare plan type, n (%)*			
Capitated or partially capitated point‐of‐service	1 (0.5)	5 (1.3)	16 (1.5)
Comprehensive	29 (14.9)	19 (4.8)	70 (6.7)
Consumer‐driven health plan	4 (2.1)	33 (8.3)	80 (7.7)
Exclusive provider organization	1 (0.5)	1 (0.3)	16 (1.5)
Health maintenance organization	15 (7.7)	50 (12.5)	91 (8.8)
High deductible health plan	7 (3.6)	15 (3.8)	69 (6.6)
Noncapitated point‐of‐service	13 (6.7)	30 (7.5)	69 (6.6)
Preferred provider organization	116 (59.8)	232 (58.1)	599 (57.6)
Unknown/missing	8 (4.1)	14 (3.5)	30 (2.9)
Total prescription burden (number of unique medications during the 6‐month period prior to DOX initiation), mean (*SD*)	9.1 (5.7)	9.5 (5.5)	9.2 (5.5)
Charlson comorbidity index score (comorbidities observed during the 6‐month period prior to DOX initiation), mean (*SD*)	6.9 (3.1)	6.2 (3.3)	6.3 (3.2)
Hypertension (ICD‐9401.x or ICD‐10 I10.x observed during the 6‐month period prior to DOX initiation), *n* (%)	77 (39.7)	159 (39.8)	389 (37.4)

Abbreviations: DOX, doxorubicin; ICD, international classification of disease; SD, standard deviation.

The most common regimens observed by group are presented in Table 2. Most patients (86.7%) received doxorubicin monotherapy in the BOL group, but only 20.6% of those in the CIV group received doxorubicin monotherapy. The most common regimen received by patients in the CIV group was doxorubicin plus ifosfamide (40.6%), whereas only 4.6% of patients received this regimen in the BOL group.

**TABLE 2:** Most commonly observed regimens at the time of doxorubicin initiation^a^.

Regimen, *n* (%)	Bolus administration (*N* = 194)	Continuous infusion (*N* = 399)	All other patients (*N* = 1040)
Doxorubicin monotherapy	170 (87.6)	82 (20.6)	184 (17.7)
Doxorubicin + ifosfamide	9 (4.6)	162 (40.6)	293 (28.2)
Doxorubicin + cisplatin	2 (1.0)	23 (5.8)	63 (6.1)
Doxorubicin + dacarbazine	1 (0.5)	58 (14.5)	74 (7.1)
Doxorubicin + olaratumab	5 (2.6)	14 (3.5)	104 (10.0)
Doxorubicin + cyclophosphamide + vincristine, etoposide + ifosfamide	2 (1.0)	0 (0.0)	74 (7.1)
Doxorubicin + docetaxel + gemcitabine	0 (0.0)	14 (3.5)	4 (0.4)

^a^ Limited to regimens used by >2% of patients in at least one group.

### 3.1 Unadjusted analyses

**FIGURE 2:**
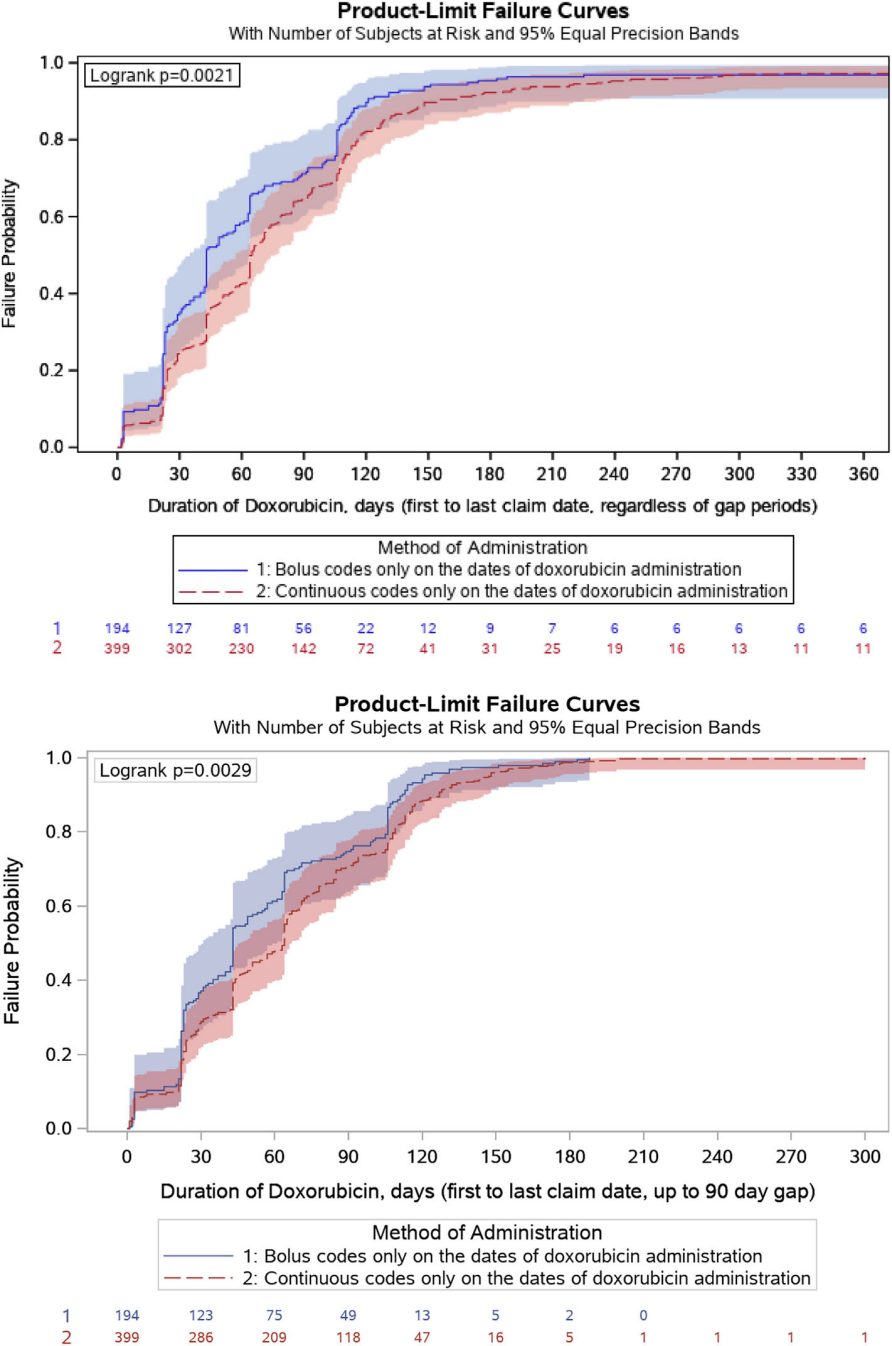
Duration of doxorubicin treatment by mode of doxorubicin administration, unadjusted analysis. (A) (top): Primary analysis ignoring gap periods. (B) (bottom): Sensitivity analysis up to the first 90‐day gap.

**FIGURE 3:**
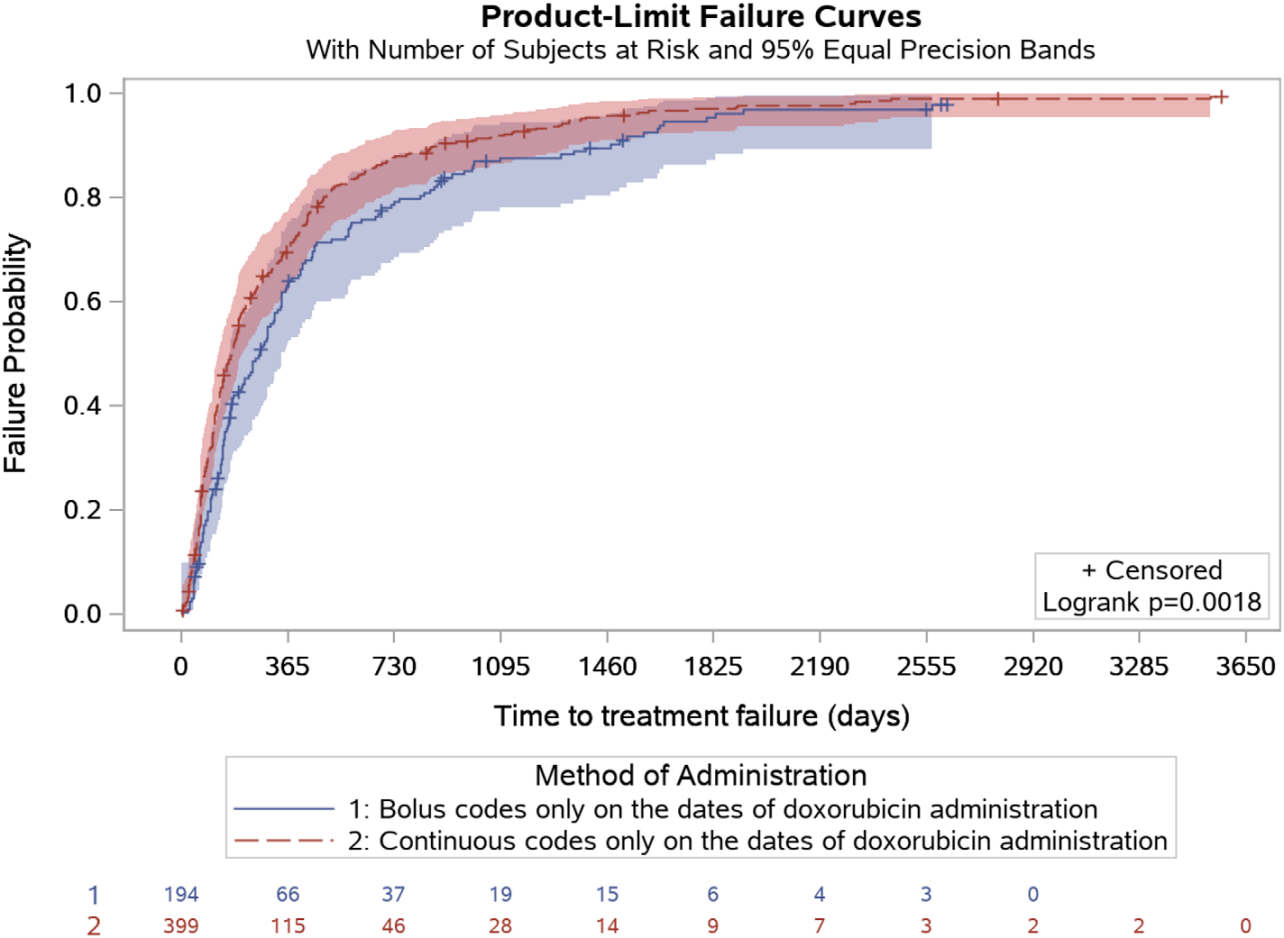
Time to treatment failure by mode of doxorubicin infusion, unadjusted analysis.

The duration of doxorubicin treatment was significantly different when comparing BOL and CIV in unadjusted analyses (*p* = .002, unadjusted log‐rank test, Figure 2A), with a median duration of treatment of 1.4 months (95% CI: 1.4–1.9) for BOL and 2.1 months (95% CI: 2.1–2.3) for CIV. When accounting for gap periods in the sensitivity analysis, these differences remained statistically significant and the median values remained consistent (1.4 months for BOL vs. 2.1 months for CIV; *p* = .003, Figure 2B). There was a slight correlation between the number of doxorubicin infusions and TTF (Pearson's correlation coefficient = 0.17, *p* < .001). Median TTF was 8.8 months for BOL (95% CI: 6.5–10.5) vs. 5.6 months for CIV (95% CI: 4.7–6.3) (*p* = .002, Figure 3).

**FIGURE 4:**
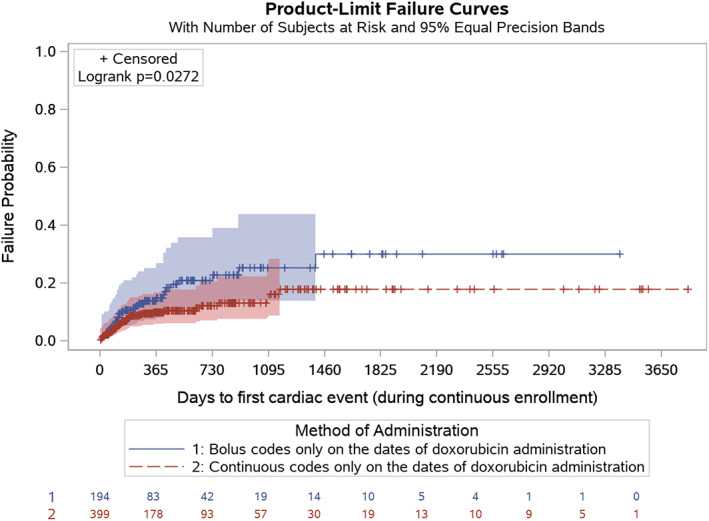
Time to first cardiac event by mode of doxorubicin infusion, unadjusted analysis.

There were no differences in cardiac outcomes for the early, middle or late time periods after doxorubicin initiation by mode of administration (Table 3). However, when considering the entire follow‐up period, there were significant differences in the incidence of cardiac events in unadjusted analyses (*p* = .03). There was also no relationship between the cumulative number of doxorubicin claims and observation of cardiac events in the early, middle, or late periods (*p* = .81, *p* = .26, and *p* = .39, respectively) but there remained no relationship between the number of doxorubicin claims and cardiac events (*p* = .75). There were significant differences in unadjusted analysis of TCE by mode of administration (*p* = .03, unadjusted log‐rank test, Figure 4). Notably, censoring was high (more than 80% in both groups) due to the relatively low number of cardiac events observed (32 events for BOL and 41 for CIV during the entire follow‐up period); the median was not reached in either group.

**TABLE 3:** Cardiac events by mode of doxorubicin administration, unadjusted analysis.

		Mode of administration		
		Bolus (*N* = 196)	Continuous (*N* = 399)	
		*n* (%)	*n* (%)	*p*‐value
Early period cardiac event codes present (≤365 days post index)	No	174 (88.8)	369 (92.5)	0.132^a^
	Yes	22 (11.2)	30 (7.5)	
Middle period cardiac event codes present (366–1825 days post index)	No	181 (92.3)	383 (96.0)	0.060^a^
	Yes	15 (7.7)	16 (4.0)	
Late period cardiac event codes present (>1825 days post index)	No	194 (99.0)	397 (99.5)	0.602^b^
	Yes	2 (1.0)	2 (0.5)	
Cardiac event present at any time during the follow‐up period	No	164 (83.7)	358 (89.7)	0.034^a^
	Yes	32 (16.3)	41 (10.3)	

^a^ Chi‐square test.

^b^ Fishers exact test.

Without considering mode of administration, there was no difference in number of doxorubicin administrations between patients with and without cardiac events for any time period evaluated, and there were no differences in duration of doxorubicin administration for all periods except for the middle period (Table 4). Median values were not reached in either group (Figure 4).

**TABLE 4:** Cardiac event by number of doxorubicin infusions and time to treatment discontinuation, unadjusted analysis.

Time period		Number of infusions		*p*‐value^a^	Time to treatment discontinuation^b^		*p*‐value^c^
		N	Mean (*SD*) infusions		Median days (95% CI)	HR^c^ (95% CI)	
Early period (≤365 days post index)	Patients with no cardiac events	1572	6.0 (4.6)	0.81	69.0 (65.0, 72.0)		
	Patients with ≥1 cardiac event	142	5.9 (4.4)		73.5 (63.0, 86.0)	1.04 (0.88, 1.23)	0.65
Middle period (366–1825 days post index)	Patients with no cardiac events	1622	6.0 (4.5)	0.26	67.0 (65.0, 71.0)		
	Patients with ≥1 cardiac event	92	6.5 (5.0)		106.0 (85.0, 108.0)	0.72 (0.58, 0.88)	0.002
Late period (>1825 days post index)	Patients with no cardiac events *N*	1706	6.0 (4.6)	0.39	69.0 (65.0, 72.0)		
	Patients with ≥1 cardiac event	8	7.4 (5.5)		94.5 (22.0, 113.0)	0.94 (0.47, 1.87)	0.85
Throughout the follow‐up period	Patients with no cardiac events *N*	1499	6.0 (4.6)	0.75	66.0 (65.0, 71.0)		
	Patients with ≥1 cardiac event	215	6.1 (4.5)		85.0 (72.0, 97.0)	0.90 (0.78, 1.03)	0.13

Abbreviations: CI, confidence interval; HR, hazard ratio; SD, standard deviation.

^a^ T‐test.

^b^ Kaplan Meier estimates.

^c^ Cox proportional hazards regression model.

### 3.2 Adjusted analyses

Stepwise variable selection was used to identify baseline variables to be retained in adjusted models. In the adjusted model for duration of doxorubicin treatment, receipt of combination therapy and codes for cerebrovascular disease, rheumatic disease, renal disease, and Kaposi's sarcoma were retained. After adjusting for these covariates, there was no difference in duration of doxorubicin treatment between BOL and CIV (HR: 1.04, 95% CI: 0.84–1.30, *p* = .71). In sensitivity analyses considering duration of treatment until the first 90‐day gap, adjusted analyses similarly found no difference in duration of therapy between BOL and CIV (HR: 1.12, 95% CI: 0.90–1.39, *p* = .33).

For TTF, insurance plan type, cerebrovascular disease, metastatic disease, diabetes with chronic complications, AIDS/HIV, or Kaposi's sarcoma codes were retained in adjusted models. After making such adjustment, there remained a statistically significant difference in TTF favoring BOL over CIV (HR = 0.71, 95% CI: 0.59–0.86, *p* = .0004).

In the adjusted model for TCE, age, metastatic codes, Kaposi's sarcoma codes, codes for congestive heart failure, and renal disease were retained as covariates (Table 5). After adjusting for these covariates, there was no significant difference in TCE by mode of administration (BOL vs. CIV: HR = 1.27, 95% CI: 0.78–2.09, *p* = .34).

**TABLE 5:** Time to cardiac event by mode of infusion (bolus vs. continuous), adjusted for covariates.

	Hazard ratio (95% confidence interval)	*p*‐value
Bolus versus continuous infusion	1.27 (0.78–2.09)	0.34
Age	1.04 (1.02–1.07)	0.0003
Congestive heart failure	3.10 (1.31–7.33)	0.01
Renal disease	2.37 (1.17–4.80)	0.02
Metastatic disease	2.50 (1.42–4.40)	0.002
Kaposi sarcoma codes	0.21 (0.07–0.59)	0.003

## 4 DISCUSSION

This real‐world database study saw statistically significant differences in unadjusted analyses. However, after adjusting for baseline covariates, there was no evidence of differences in the risk of cardiac events linked to doxorubicin treatment for sarcoma by mode of infusion (BOL vs. CIV). The differences observed in the unadjusted analyses were therefore associated with those baseline factors, rather than the method of infusion. Once the confounding factors were accounted for, this study suggests that a mitigation strategy based on replacing BOL administration with a prolonged continuous infusion may not result in the hoped‐for reduction in cardiotoxicity, based on the community standards for use of doxorubicin in sarcoma treatment. Fortunately, cardiac events were uncommon in both treatment groups.

These findings are consistent with preliminary analyses from SARC021, a large randomized trial comparing doxorubicin with or without evofosfamide.^20^ While this was an unplanned *post hoc* analysis, the investigators found no relationship between cardiac adverse event outcomes for BOL versus CIV. Thus, both SARC021 trial and the current retrospective database study consistently failed to find value in the substitution of CIV for BOL doxorubicin administration for the purposes of reducing cardiotoxicity when caring for patients with sarcoma. Since CIV administration of doxorubicin is proposed to mitigate potential harm of anthracycline therapy, our data are unable to find evidence of harm in using the logistically more favorable BOL administration strategy in sarcoma treatment.

This conclusion is qualified by the previously noted doxorubicin dosing limitations typically imposed. Higher doses of doxorubicin may be associated with better clinical outcomes from a sarcoma control standpoint.^24^, ^25^ If CIV allows such administration, then there may be harm to patients through non‐receipt of more aggressive doxorubicin dosing. CIV administration studies have instituted the CIV intervention from treatment initiation. The cohort we selected should similarly receive CIV doxorubicin from initiation of treatment, as patients receiving doxorubicin with both CIV and BOL codes were excluded from the study. Perhaps delaying institution of CIV until the 300 mg/m^2^ threshold is reached, would allow better acceptance of CIV administration as an intervention, focusing it on those patients more likely to benefit.^7^ That being said, evidence of cardiac effects from doxorubicin can be identified within hours of first doxorubicin treatment.^26^ This perhaps argues for earlier intervention, rather than using a threshold defined by the probability of clinical cardiac toxicity, likely an insensitive measure of cardiac effects. Balancing these competing considerations is beyond the scope of this study.

The MarketScan insurance claims database used in this study was not intended to address the questions posed herein. To use this dataset, appropriate patient populations and endpoints needed to be identified. This required the use of surrogate markers to reflect more conventional parameters. For example, TTF was one of the major efficacy endpoint under study, yet was not a specific datapoint available in the MarketScan database. Instead, we developed a composite definition (see methods/outcomes evaluated, above). Patients either started a new therapy, enrolled in hospice care, or had a final data entry time point (in the latter case leading to censoring in the time‐to‐event analyses). While imperfect, we believe such a commonsense definition reasonably reflects “treatment failure,” allowing TTF to be assessed.

Selection of appropriate cohorts for study was also important, and required adaptation to the nature of information available in MarketScan. As cardiac toxicity was of major interest, and no individual cardiac assessments data (such as echocardiography results) were available, other criteria were required to exclude those with pre‐existing cardiac dysfunction. While it would be hoped that this would be undertaken as part of routine clinical care prior to doxorubicin treatment, we did not assume this. Thus, patients with a significant pre‐existing history of heart disease, as determined by linkage in the database with diagnostic codes for cardiac conditions (specifically, at least two outpatient codes or one inpatient code) were excluded from the study cohort. Patients linked to a single outpatient cardiovascular disease diagnostic code could be included. The CCI also incorporates several cardiovascular conditions.^23^ Interestingly, adjusted Cox regression models retained congestive heart failure and cerebrovascular disease as covariates, even though very few patients were identified with one of these codes. This difference is likely due to the study criteria requiring two or more cardiac codes, whereas the CCI is less stringent. The observation of one or more codes indicates the presence of a comorbid condition on the CCI. Therefore, the comorbidity covariates included presence of at least one code for congestive heart failure and cerebrovascular disease, both of which were retained in the final models. While very few patients reported these codes, likely due to the exclusion criteria for the study for two or more codes, these covariates nevertheless were retained in adjusted models developed using stepwise regression. While causal relationships cannot be inferred due to lack of randomization, these data support the presumption that patients with a history of these factors may be at increased risk of doxorubicin‐associated cardiac events.

Despite there being no differences in the duration of treatment between BOL and CIV in adjusted analyses, there remained significant differences in TTF by mode of administration, after adjusting for baseline covariates. Patients receiving BOL doxorubicin had a significantly longer duration of time until the initiation of a subsequent therapy than those receiving CIV doxorubicin. TTF has been evaluated as a proxy for overall survival in advanced sarcomas; while imperfect for that purpose, it has value as a marker of clinical outcomes.^27^

It is possible that patients with less favorable prognoses were preferentially selected to receive multi‐agent chemotherapy. Although monotherapy versus multi‐agent therapy was not a retained variable in adjusted TTF models, metastatic disease was retained, possibly serving as the prognostic indicator. The line of therapy in which doxorubicin was initiated may influence TTF, but was not considered in this study. Similarly, the utilization of dexrazoxane as a cardioprotectant and type of post doxorubicin therapy were not evaluated.

Cardiac event failure probability was higher in BOL‐treated patients than in CIV in unadjusted analyses (Figure 4). However, in adjusted analyses, mode of treatment administration was not significant (Table 5). Instead, increasing age, congestive heart failure, renal disease, and metastatic disease were associated with a higher cardiac event probability. A code for Kaposi's sarcoma was, in contrast, protective against development of a cardiac event.

Strategies other than altering manner of doxorubicin administration are available to mitigate anthracycline cardiac risk. These potentially include novel anthracycline derivatives with less cardiotoxicity (e.g. epirubicin), liposomal encapsulation,^9^, ^10^, ^11^, ^12^, ^13^ and improving the use of dexrazoxane, a cardioprotective agent in combination with anthracyclines.^7^, ^8^

In a recent study in which up to 8 cycles of doxorubicin (equivalent to 600 mg/m^2^) were administered with concurrent use of the dexrazoxane cardioprotective agent, cardiac toxicity was low (1%–3%) regardless of the cumulative dose administered, suggesting that dexrazoxane could be used to mitigate cardiac risk among patients with sarcoma.^25^ Interim results of a prospective, single‐arm phase II trial, in which sarcoma patients receive dexrazoxane with doxorubicin from treatment initiation, support this finding.^24^ While these studies also suggest that more aggressive treatment with doxorubicin was possible, and might be associated with increased clinical benefit, their findings remain preliminary, and access to dexrazoxane itself may be limited in treatment of sarcoma patients internationally, perhaps due to cost, regulatory hurdles, or lack of familiarity with the data.

As with all retrospective claims database studies, there are limitations with ICD coding and the ability to identify patients with sarcoma. This was overcome, in part, by requiring doxorubicin use in this cohort. It is still possible that some patients with sarcoma were coded under other disease sites and consequently excluded.^28^ This could affect the generalizability of our findings. Additionally, most patients could not be accurately categorized into a BOL or CIV infusion group due to utilization of multiple codes. Instead of imputation or algorithmic logic to increase sample size, the study team intentionally limited the cohorts to patients with clear coding to ensure the groups truly represented those who received doxorubicin by either mode. The exclusion of any patient whose infusion coding was unclear, while markedly reducing sample size, retained the ability to make clean comparisons between groups. Generalizability was reduced to ensure internal validity of group assignment to answer more accurately the primary research questions regarding the impact of BOL versus CIV. Specifically, the conclusions drawn from this work show that overall there are no differences in cardiac toxicities by method of doxorubicin administration, after adjusting for baseline covariates. However, the conclusions cannot be assumed for all specific regimens, doses or schedules of infusion. As specific regimens that used multiple codes could not be defined as either BOL or CIV, caution should be made in the inference of these findings to the outcomes of specific treatment strategies.

Other key variables are not present in the claims dataset that would have enhanced this analysis. Lack of exact doxorubicin dose prohibited the analysis of cumulative dose effects. The total number of doxorubicin claims was used as a surrogate, as each infusion would be associated with a single claim. Therefore, the number of doxorubicin infusions should be equivalent to the number of claims, but this could not be verified with certainty. We assumed that a greater number of doxorubicin claims was most likely associated with higher cumulative doses. The lack of relationship between number of doxorubicin claims and cardiac events may indicate that the relationship is likely not linear, as cumulative dose has been long known to be associated with cardiac events, or that the study lacked the power to detect a difference in the two administration groups.^4^ However, with 194 BOL and 399 CIV patients, the study cohorts herein are significantly larger than those participating in earlier randomized studies assessing impact of administration route.

## 5 CONCLUSIONS

The evidence from this study suggests that using CIV in place of BOL administration of doxorubicin may not be associated with a reduction in cardiotoxicity. Despite the known limitations of retrospective database studies depending upon diagnostic and procedural coding, the findings are consistent with the emerging evidence that CIV does not mitigate the risk of doxorubicin‐associated cardiotoxicity, when doxorubicin is administered according to the general oncologic practices within the source population. This presumably would include a generally accepted limitation of the cumulative doxorubicin dose to be received by a given patient.^4^, ^5^ As BOL administration appears to have been adopted widely in sarcoma treatment, it is reassuring that this method does not appear to be harmful versus CIV in either increasing cardiac toxicity or reducing treatment efficacy.

## AUTHOR CONTRIBUTIONS

**Lee D. Cranmer:** Conceptualization (equal); resources (supporting); visualization (equal); writing – original draft (supporting); writing – review and editing (equal). **Lisa M. Hess:** Conceptualization (equal); data curation (supporting); formal analysis (lead); investigation (equal); methodology (lead); resources (equal); visualization (equal); writing – original draft (lead); writing – review and editing (equal). **Tomoko Sugihara:** Data curation (lead); formal analysis (lead); investigation (equal); methodology (supporting); software (lead); validation (lead); writing – original draft (supporting); writing – review and editing (supporting). **Howard G. Muntz:** Conceptualization (equal); formal analysis (supporting); methodology (supporting); writing – original draft (supporting); writing – review and editing (supporting).

## FUNDING INFORMATION

This was an unfunded study conducted with material support from Eli Lilly and Company.

## CONFLICT OF INTEREST

The authors declare no conflicts of interest.

## ETHICS STATEMENT

This study involved no human subjects and relied exclusively on de‐identified data.

## Data Availability

Data used in this study were supplied by International Business Machines (IBM) Corporation; these databases are available directly by licensing from IBM. Any analysis, interpretation, or conclusion based on these data is solely that of the authors and not International Business Machines Corporation.

## References

[1] Judson I , Verweij J , Gelderblom H , et al. Doxorubicin alone versus intensified doxorubicin plus ifosfamide for first‐line treatment of advanced or metastatic soft‐tissue sarcoma: a randomised controlled phase 3 trial. Lancet Oncol. 2014;15(4):415‐423. 24618336 10.1016/S1470-2045(14)70063-4

[2] Ryan CW , Merimsky O , Agulnik M , et al. PICASSO III: a phase III, placebo‐controlled study of doxorubicin with or without Palifosfamide in patients with metastatic soft tissue sarcoma. J Clin Oncol. 2016;34(32):3898‐3905. 27621408 10.1200/JCO.2016.67.6684

[3] Tap WD , Papai Z , van Tine BA , et al. Doxorubicin plus evofosfamide versus doxorubicin alone in locally advanced, unresectable or metastatic soft‐tissue sarcoma (TH CR‐406/SARC021): an international, multicentre, open‐label, randomised phase 3 trial. Lancet Oncol. 2017;18(8):1089‐1103. 28651927 10.1016/S1470-2045(17)30381-9 PMC7771354

[4] von Hoff DD , Layard MW , Basa P , et al. Risk factors for doxorubicin‐lnduced congestive heart failure. Ann Intern Med. 1979;91(5):710‐717. 496103 10.7326/0003-4819-91-5-710

[5] Minow RA , Benjamin RS , Lee ET , Gottlieb JA . Adriamycin cardiomyopathy—risk factors. Cancer. 1977;39(4):1397‐1402. 856438 10.1002/1097-0142(197704)39:4<1397::aid-cncr2820390407>3.0.co;2-u

[6] Babiker HM , McBride A , Newton M , et al. Cardiotoxic effects of chemotherapy: a review of both cytotoxic and molecular targeted oncology therapies and their effect on the cardiovascular system. Crit Rev Oncol Hematol. 2018;126:186‐200. 29759560 10.1016/j.critrevonc.2018.03.014

[7] Seymour L , Bramwell V , Moran LA . Use of dexrazoxane as a cardioprotectant in patients receiving doxorubicin or epirubicin chemotherapy for the treatment of cancer. The provincial systemic treatment disease site group. Cancer Prev Control. 1999;3(2):145‐159. 10474762

[8] Liesse K , Harris J , Chan M , Schmidt ML , Chiu B . Dexrazoxane significantly reduces anthracycline‐induced cardiotoxicity in pediatric solid tumor patients: a systematic review. J Pediatr Hematol Oncol. 2018;40(6):417‐425. 29432315 10.1097/MPH.0000000000001118 PMC6059999

[9] Hortobagyi GN , Yap HY , Kau SW , et al. A comparative study of doxorubicin and epirubicin in patients with metastatic breast cancer. Am J Clin Oncol. 1989;12(1):57‐62. 2643296 10.1097/00000421-198902000-00014

[10] Perez DJ , Harvey VJ , Robinson BA , et al. A randomized comparison of single‐agent doxorubicin and epirubicin as first‐line cytotoxic therapy in advanced breast cancer. J Clin Oncol. 1991;9(12):2148‐2152. 1960557 10.1200/JCO.1991.9.12.2148

[11] Smith LA , Cornelius VR , Plummer CJ , et al. Cardiotoxicity of anthracycline agents for the treatment of cancer: systematic review and meta‐analysis of randomised controlled trials. BMC Cancer. 2010;10:337. 20587042 10.1186/1471-2407-10-337 PMC2907344

[12] Mouridsen HT , Bastholt L , Somers R , et al. Adriamycin versus epirubicin in advanced soft tissue sarcomas. A randomized phase II/phase III study of the EORTC soft tissue and bone sarcoma group. Eur J Cancer Clin Oncol. 1987;23(10):1477‐1483. 3479329 10.1016/0277-5379(87)90089-7

[13] Nielsen OS , Dombernowsky P , Mouridsen H , et al. High‐dose epirubicin is not an alternative to standard‐dose doxorubicin in the treatment of advanced soft tissue sarcomas. A study of the EORTC soft tissue and bone sarcoma group. Br J Cancer. 1998;78(12):1634‐1639. 9862576 10.1038/bjc.1998.735 PMC2063236

[14] Bielack SS , Erttmann R , Winkler K , Landbeck G . Doxorubicin: effect of different schedules on toxicity and anti‐tumor efficacy. Eur J Cancer Clin Oncol. 1989;25(5):873‐882. 2661240 10.1016/0277-5379(89)90135-1

[15] Legha SS , Benjamin RS , Mackay B , et al. Adriamycin therapy by continuous intravenous infusion in patients with metastatic breast cancer. Cancer. 1982;49(9):1762‐1766. 7074581 10.1002/1097-0142(19820501)49:9<1762::aid-cncr2820490905>3.0.co;2-q

[16] Legha SS , Benjamin RS , Mackay B , et al. Reduction of doxorubicin cardiotoxicity by prolonged continuous intravenous infusion. Ann Intern Med. 1982;96(2):133‐139. 7059060 10.7326/0003-4819-96-2-133

[17] Shapira J , Gotfried M , Lishner M , Ravid M . Reduced cardiotoxicity of doxorubicin by a 6‐hour infusion regimen. A prospective randomized evaluation. Cancer. 1990;65(4):870‐873. 2297656 10.1002/1097-0142(19900215)65:4<870::aid-cncr2820650407>3.0.co;2-d

[18] Zalupski M , Metch B , Balcerzak S , et al. Phase III comparison of doxorubicin and dacarbazine given by bolus versus infusion in patients with soft‐tissue sarcomas: a Southwest Oncology Group study. J Natl Cancer Inst. 1991;83(13):926‐932. 2067035 10.1093/jnci/83.13.926

[19] Casper ES , Gaynor JJ , Hajdu SI , et al. A prospective randomized trial of adjuvant chemotherapy with bolus versus continuous infusion of doxorubicin in patients with high‐grade extremity soft tissue sarcoma and an analysis of prognostic factors. Cancer. 1991;68(6):1221‐1229. 1873773 10.1002/1097-0142(19910915)68:6<1221::aid-cncr2820680607>3.0.co;2-r

[20] Cranmer LD , Lu Y , Ballman KV , Loggers ET , Pollack SM . Toxicity and efficacy of bolus (BOL) versus continuous intravenous (CIV) dosing of doxorubicin (DOX) in soft tissue sarcoma (STS): post hoc analysis of a prospective randomized trial. J Clin Oncol. 2017;35:11023. 10.1158/1078-0432.CCR-22-1564 36622694

[21] IBM . IBM Marketscan research databases for life sciences researchers 2021 https://www.ibm.com/downloads/cas/OWZWJ0QO.

[22] HHS . Human subject regulations decision charts: 2018 https://www.hhs.gov/ohrp/regulations-and-policy/decision-charts-2018/index.html#c1.

[23] Glasheen WP , Cordier T , Gumpina R , Haugh G , Davis J , Renda A . Charlson comorbidity index: ICD‐9 update and ICD‐10 translation. Am Health Drug Benefits. 2019;12(4):188‐197. 31428236 PMC6684052

[24] van Tine BA , Hirbe AC , Oppelt P , et al. Interim analysis of the phase II study: noninferiority study of doxorubicin with upfront dexrazoxane plus olaratumab for advanced or metastatic soft‐tissue sarcoma. Clin Cancer Res. 2021;27(14):3854‐3860. 33766818 10.1158/1078-0432.CCR-20-4621 PMC8282681

[25] Jones RL , Wagner AJ , Kawai A , et al. Prospective evaluation of doxorubicin cardiotoxicity in patients with advanced soft‐tissue sarcoma treated in the ANNOUNCE phase III randomized trial. Clin Cancer Res. 2021;27:3861‐3866. 33632930 10.1158/1078-0432.CCR-20-4592 PMC8282740

[26] Unverferth BJ , Magorien RD , Balcerzak SP , Leier CV , Unverferth DV . Early changes in human myocardial nuclei after doxorubicin. Cancer. 1983;52(2):215‐221. 6861067 10.1002/1097-0142(19830715)52:2<215::aid-cncr2820520206>3.0.co;2-f

[27] Savina M, Litiere S, Italiano A, et al. Surrogate endpoints in advanced sarcoma trials: a meta analysis. *Oncotarget*. 2018;9(77):34617‐34627. 10.18632/oncotarget.26166 PMC6195375 30349653

[28] Hess LM , Zhu YE , Sugihara T , Fang Y , Collins N , Nicol S . Challenges of using ICD‐9‐CM and ICD‐10‐CM codes for soft‐tissue sarcoma in databases for health services research. Perspect Health Inf Manag. 2019;16:1‐13. PMC6462881 31019431

